# Cancer risk among people living with HIV in Rwanda from 2007 to 2018

**DOI:** 10.1002/ijc.35091

**Published:** 2024-08-11

**Authors:** Jean Claude Dusingize, Gad Murenzi, Benjamin Muhoza, Lydia Businge, Eric Remera, Francois Uwinkindi, Marc Hagenimana, Gallican Rwibasira, Sabin Nsanzimana, Philip E. Castle, Kathryn Anastos, Gary Clifford

**Affiliations:** 1 Cancer Epidemiology, Prevention & Control Program, Montefiore Einstein Cancer Center.; 2 Einstein-Rwanda Research and Capacity Building Program, Research for Development (RD Rwanda), Kigali, Rwanda.; 3 Rwanda Biomedical Centre, Kigali, Rwanda.; 4 The Ministry of Health of Rwanda, Kigali, Rwanda; 5 Divisions of Cancer Prevention and Cancer Epidemiology and Genetics, National Cancer Institute, Bethesda, Maryland, USA; 6 Departments of Medicine and of Epidemiology & Population Health, Albert Einstein College of Medicine, Bronx, New York, USA.; 7 Early Detection Prevention and Infections Branch, International Agency for Research on Cancer, Lyon, France.

**Keywords:** HIV infection, cancer, data linkage, epidemiology

## Abstract

Assessing the risk of cancer among people living with HIV (PLHIV) in the current era of antiretroviral therapy (ART) is crucial, given their increased susceptibility to many types of cancer and prolonged survival due to ART exposure. Our study aims to compare the association between HIV infection and specific cancer sites in Rwanda. Population-based cancer registry data were used to identify cancer cases in both PLHIV and HIV-negative persons. A probabilistic record linkage approach between the HIV and cancer registries was used to supplement HIV status ascertainment in the cancer registry. Associations between HIV infection and different cancer types were evaluated using unconditional logistic regression models. We performed several sensitivity analyses to assess the robustness of our findings and to evaluate the potential impact of different assumptions on our results. From 2007 to 2018, the cancer registry recorded 17,679 cases, of which 7% were diagnosed among PLHIV. We found significant associations between HIV infection and Kaposi’s Sarcoma (KS) (adjusted odds ratio (OR)): 29.1, 95% CI: 23.2–36.6), non-Hodgkin lymphoma (NHL) (1.6, 1.3–2.0), Hodgkin lymphoma (HL) (1.6, 1.1–2.4), cervical (2.3, 2.0–2.7), vulvar (4.0, 2.5–6.5), penile (3.0, 2.0–4.5) and eye cancers (2.2, 1.6–3.0). Men living with HIV had a higher risk of anal cancer (3.1, 1.0–9.5) than men without HIV, but women living with HIV did not have higher risk than women without HIV (1.0, 0.2–4.3). Our study found that in an era of expanded ART coverage in Rwanda, HIV is associated with a broad range of cancers, particularly those linked to viral infections.

## Graphical Abstract

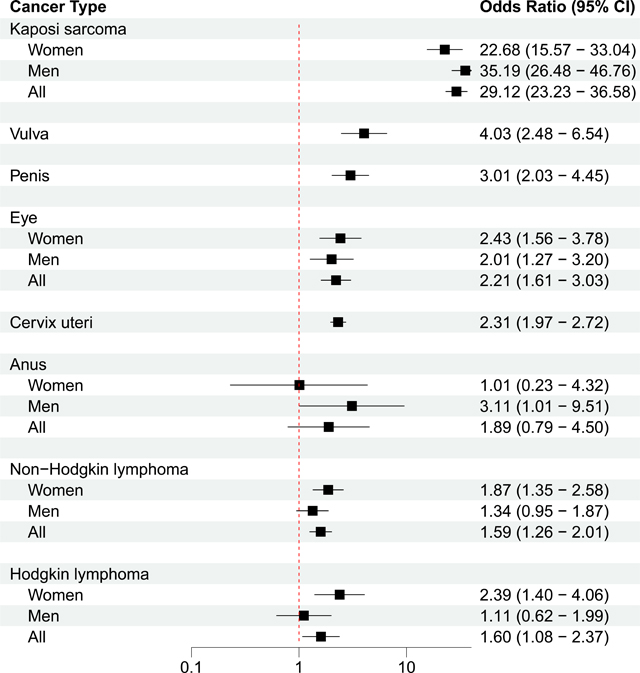

## Introduction

Sub-Saharan Africa (SSA) comprises only 12% of the world population ^1^, yet it is home to more than 70% of the estimated 38 million people living with human immunodeficiency virus (PLHIV) globally ^2^. The advent of antiretroviral therapy (ART) has substantially changed the course of the epidemic by improving immune function and reducing mortality related to opportunistic infections ^3^. With ART-driven improvements in life-expectancy, however, cancer has become one of the leading causes of mortality in PLHIV ^4, 5^.

Elevated risks of cancer in PLHIV are believed to be largely driven by increased exposure to infectious carcinogens, acquired through shared transmission routes, combined with the acceleration of viral carcinogenesis by HIV-induced immunosuppression ^6, 7^. Indeed, almost all malignancies occurring at excess risk in PLHIV are linked to co-infection with oncogenic viruses, most notably Epstein-Bar virus (EBV), human herpesvirus 8 (HHV-8), and human papillomavirus (HPV) ^6^.

Furthermore, cancer types occurring among PLHIV are shifting over time and vary in their relative burden in different parts of the world. While the risk of cancer among PLHIV has been extensively evaluated in high-income countries ^8–10^, data on those malignancies in SSA are limited. The scarcity of high-quality cancer registry data ^11^ and small sample sizes in hospital-based studies have restricted research efforts in this region. A prior study reported cancer registry data by HIV status from Rwanda; however, it was descriptive in nature and did not assess the epidemiological association between HIV and specific cancer sites ^12^.

Rwanda has invested heavily in building data infrastructures and in cancer diagnosis and management to deal with a growing cancer burden. In collaboration with the International Agency for Research on Cancer (IARC) and the US National Institutes of Health (NIH), a population-based cancer registry was developed with active collection of cases nationwide. Rwanda also has one of the most well-developed HIV testing and treatment programs in low-income countries, which is a key factor in the country’s success in ensuring comprehensive nationwide access to ART since 2005 ^13, 14^. For example, at the end of 2009, 77% of adults in need of ART were receiving treatment ^15^ and this percentage rose to 92% in 2022 ^16^. Currently, the prevalence of HIV infection among adults aged 15–49 in Rwanda is approximately 3% ^17^. Given such widespread testing and knowledge of HIV status, particularly at time of cancer diagnosis at tertiary hospitals, cancer registries in Rwanda increasingly report on HIV status. Leveraging these existing infrastructures, we employed a probabilistic record linkage approach between the HIV and cancer registries to supplement the ascertainment of HIV status in the cancer registry and thus to estimate the cancer burden by HIV status during the ART era in Rwanda. A better understanding of the link between HIV infection and cancer risk in this population is key to guiding tailored cancer prevention efforts.

## Materials and Methods

### The population-based cancer registry in Rwanda

In 2010, Women’s Equity in Access to Care and Treatment (WE-ACTx) received a small grant to reinitiate the Rwandan Cancer Registry. In collaboration with expertise provided by the IARC, abstraction of cancer cases was performed retrospectively from 2007 to 2010 and prospectively since 2011. Cancer registrars carried out active case finding by searching for cases in different national reference hospitals, district hospitals and pathology laboratories in Rwanda (Supplementary Table 1). The cancer notification process captured essential details, including diagnosis (topography and morphology), means of diagnosis (histology, diagnostic imaging, and clinical only), basic demographic information (age, sex, and address), and the source of information. Confirmed cancer cases were classified according to their primary anatomical site of cancer (topography) and histological morphology when available using the World Health Organization (WHO) third edition of the International Classification of Diseases for Oncology (ICD-O-3) ^18^. The cancer registry also increasingly captures information on HIV status, although it is not complete.

### Supplemental linkage with HIV registry

We conducted supplementary data linkage between the cancer registry and the national HIV registry, to enhance the ascertainment of HIV status in the cancer registry. The Open Medical Record System (OpenMRS; https://openmrs.org/) is currently used for data entry and management in most healthcare facilities providing HIV care and treatment in Rwanda (hereafter referred to as the HIV registry). The linkage with the cancer registry involved a total of 140,427 participants from the HIV registry, concentrated in clinics in Kigali.

The HIV and cancer registry data were linked using family and other names or initials, date of birth and place of residence, using the Software for Automated Linkage version 3.3 (SALI3.3) ^19^. SALI allows spelling errors in names and facilitates a double-blinded procedure to visually compare the similarity of encrypted names, meaning neither the operator nor researcher can see the patients’ personal identifiers (for confidentiality reasons). SALI uses a probabilistic matching algorithm that evaluates the likelihood of subject linkage on the basis of identical or near-identical information in the two linked datasets. Probabilistic record linkage is a valid and useful tool to combine datasets without a unique patient identification number ^20^.

### Statistical analysis

Patients’ characteristics were compared according to HIV status (negative, positive, unknown) using proportions for categorical variables, or median and interquartile range (IQR) for continuous variables. Associations between HIV infection and different cancer types were evaluated using unconditional logistic regression models adjusted for age (treated as a continuous variable in the model), gender, province of residence and year of cancer diagnosis. For each cancer site, odds ratios (OR) and corresponding 95% confidence intervals (95% CI) were calculated by comparing the proportion of the given cancer in PLHIV versus that in HIV-negative participants. In final OR analyses, we considered persons with unknown HIV status to be persons without HIV, as their baseline characteristics (See Table 1) and distribution of cancers (See Table 2 and Table 3) were similar. However, we also performed sensitivity analyses including only cases with confirmed HIV status. Because our analyses compared the relative frequencies of different cancer types among PLHIV to those without HIV, all of whom were diagnosed with at least one type of cancer, cancers not associated with HIV infection were underrepresented among PLHIV. To address this, we repeated analyses after excluding all cancers significantly associated with HIV-infection (i.e., KS, NHL, HL, cervix, vulva, penis, anus, and eye cancers). Lastly, we conducted additional sensitivity analyses restricted to cancers diagnosed after 2013, as the accuracy of cancer ascertainment was limited before that time period. All statistical analyses were performed using SAS software, version 9.4 (SAS Institute, Cary, NC).

## Results

The cancer registry comprised a total of 17,679 cancer cases. Prior to data linkage between the cancer and HIV registries, 965 (5.4%) HIV positive cases were recorded in the cancer registry. After performing the linkage, 265 additional cases in the cancer registry (174 HIV unknown and 91 HIV-negative) were re-classified as HIV positive, bringing the total number of cancers in the cancer registry considered HIV positive to 1,230 (Table 1). Of note, only 77 cases (6.3%) originally considered to be HIV-positive in the cancer registry were confirmed to be HIV-positive by the linkage, but this proportion was 4-fold higher than HIV-negatives (1.6%) and HIV unknowns (1.7%). These proportions varied by cancer site and were higher for persons residing in Kigali (where the majority of study clinics contributing to the HIV registry were situated) (Supplementary Table 2).

Overall, 7% of cancer cases were diagnosed among PLHIV, 33% among HIV-negative persons, and the remaining 60% had an unknown HIV status (Table 1). Cancers diagnosed among PLHIV were diagnosed at a younger age than those who were HIV negative: median age (interquartile range) of 43 (34–50) vs. 50 (35–62) years. More cancer cases were diagnosed in females than males (60% vs. 40%). Compared to other provinces, Kigali, the capital and largest city, had the highest proportion of cancer cases diagnosed among PLHIV, accounting for 38% of cases. We observed a strong increasing trend in numbers of cancer cases diagnosed between 2007 and 2018, highlighting improvements in cancer diagnosis and registration during this period. Furthermore, most cases (~75%) had a confirmed diagnosis through histopathological examination (Table 1).

The distribution of cancer types by HIV status is shown in Table 2 for women, and in Table 3 for men. Of 10,534 cancers in women, the two most commonly diagnosed were cervical (21.7%) and breast (20.0%), irrespective of HIV status. Kaposi’s sarcoma (KS) and non-Hodgkin lymphoma (NHL) were the third and fourth most common cancers among women living with HIV (WLWH), but not HIV-negative women (Table 2). Among 7,145 cancers observed in men, prostate (13.8%) and stomach (10.5%) were the two most commonly diagnosed types overall (Table 3). Among men living with HIV (MLWH), however, KS was the most commonly diagnosed cancer type, accounting for more than a third (36.3%) of the burden, followed by NHL (9.3%). The fraction of cancer contributed by KS was higher in men than women (36.3% vs. 10.7% respectively).

**Figure 1.**
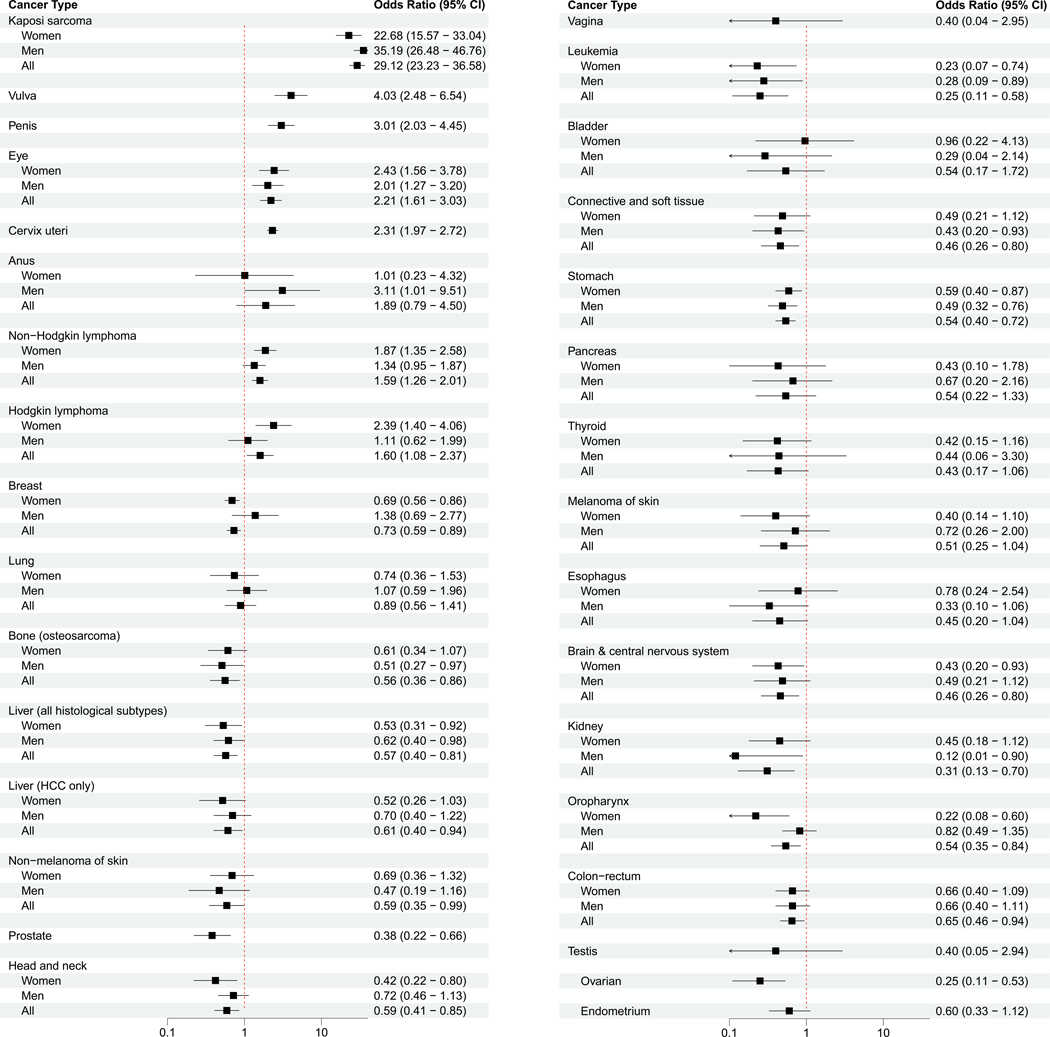
Association of HIV infection with specific cancers diagnosed in Rwanda, 2007–2018. Odds ratios are determined using logistic regression models adjusting for age, gender (where applicable), place of residence and year of cancer diagnosis. Head and neck include oropharynx.

Associations between HIV infection and cancer types, overall and by gender, are presented in Figure 1. Compared with cancer diagnosed in HIV-negative persons, PLHIV with cancer had significantly higher odds of being diagnosed with KS (OR: 29.1, 95% CI: 23.2–36.6), NHL (1.6, 1.3–2.0), Hodgkin lymphoma (HL) (1.6, 1.1–2.4), cervical (2.3, 2.0–2.7), vulvar (4.0, 2.5–6.5), penile (3.0, 2.0–4.5) and eye cancers (2.2, 1.6–3.0). MLWH were significantly more likely to be diagnosed with anal cancer (3.1, 1.0–9.5), but WLWH were not (1.0, 0.2–4.3). KS and NHL were significantly elevated in both men and women living with HIV, whereas the association between HIV and HL reached statistical significance only in women. The proportions of these cancers known to be HIV-associated are shown by age in Supplementary Table 3 and tend to be higher than those of non-HIV-associated cancers in every age group.

In a sensitivity analysis based on cases with confirmed HIV status only (i.e., after exclusion of those with unknown HIV status from the HIV-negative group), no material differences were observed, except that loss of statistical power led to associations for cancer of the anus and Hodgkin lymphoma to no longer be significant (Supplementary Table 4). We also repeated analyses of the distribution of cancer types, after excluding all cancers significantly associated with HIV-infection as mentioned above (i.e., KS, NHL, HL, cervix, vulva, penis, anus, and eye cancers). These analyses showed no significant associations by HIV status, except for breast cancer, which was over-represented among cancers diagnosed in WLWH compared to cancers among HIV-negative women (Supplementary Table 5). Finally, when restricting the analyses to cancers diagnosed from 2013, no remarkable changes were observed in the estimates, except for a decrease in statistical power to detect significant associations with anal and eye cancers among men, and a decrease in risk estimates for KS (Supplementary Table 6).

## Discussion

In this study we employed the population-based cancer registry data during the period 2007–2018 to evaluate cancer risk in PLHIV in Rwanda. Our investigation revealed that PLHIV had elevated risk of developing a wide spectrum of cancers compared to HIV-negative persons, particularly those known to be associated with viral infections. The increased risk of multiple cancer types observed in our study underscores the fact that in an era of high ART coverage a broad-spectrum of cancer in Rwanda may be attributable to HIV. Furthermore, our research indicates that even for cancer types that are well established to be linked to HIV infection, HIV testing and reporting rates among cancer patients in this population remain sub-optimal.

Our study confirmed the strong and established association between KS and HIV infection in SSA, in both men and women ^21, 22^. The strength of our relative risk estimate for KS (~30) was notably lower compared to that reported in high-income settings (where it reaches >1,000) ^10, 23^. However, prior to the HIV epidemic, whereas KS was relatively rare in these high-income settings ^24^, it was endemic in SSA, particularly among men ^25^. The lower relative risk of KS observed in our study, and others in sub-Saharan African settings ^22, 26^, therefore, is expected to partially reflect a higher KS background risk existing among HIV-negative persons. Indeed, KS accounted for 0.3% of cancers in HIV-negative persons in our study, whereas KS represents only 0.05% of all cancers in the U.S.A. population (including those occurring in HIV-positive persons). On the other hand, our estimate of 63% of KS among persons known to be HIV-positive was somewhat lower than the 82% reported in a previous hospital-based case-control study in Rwanda ^27^ and the approximately 80% reported in other sub-Saharan African settings ^28^, which may be due to some misclassification of HIV positivity in KS among persons of unknown HIV status.

Our study contributes to the growing body of evidence highlighting the significant impact of HIV on NHL in SSA. Linkage studies conducted in South Africa and Uganda have shown that NHL incidence was significantly higher among PLHIV compared to HIV-negative persons ^21, 22^. Our study also revealed a higher relative risk of Hodgkin lymphoma (HL) among WLWH, but that was not confirmed among men. The risk of HL associated with HIV has not been previously reported by sex; thus this potential sex difference merits further investigation.

Our study confirms the well-established association between HIV infection and cervical cancer risk ^627, 29^. We observed an increase in the odds of other HPV-related anogenital cancers, including vulva, anus and penis cancers, consistent with previous studies conducted in SSA ^22, 27, 30^, and a recent meta-analysis of cohort and registry linkage studies, predominantly from high-income settings^31^. It is noteworthy that in the latest IARC monograph, the evidence linking HIV infection with vulvar and penile cancers was still considered limited ^6^. Additionally, our study confirms the well-established association between HIV infection and anal cancer. Notably, the odds ratio for anal cancer was higher in men than women, potentially reflecting differences in anal HPV transmission between men and women in SSA ^32–34^. We did not find significant evidence to establish a relationship between HIV infection and the risk of vaginal cancer, consistent with previous studies conducted in SSA ^22, 27^. Other studies combined data for anogenital organs other than cervix ^30, 35^.

There is also a growing body of evidence linking HPV infection to the risk of developing certain head and neck cancers, particularly oropharyngeal squamous cell carcinoma ^36–38^. Although evidence from registry-based linkage studies in Switzerland and USA suggests that PLHIV have an elevated risk of developing oropharyngeal cancer compared to the general population ^39, 40^, this is partially attributed to the higher prevalence of smoking among PLHIV ^41, 42^. Of note, our study found no evidence that head and neck cancers were associated with HIV infection (nor with lung cancer that suffers from a similar tobacco confounding issue).

Our study found a strong association between HIV infection and eye cancer, with SCC of the conjunctiva being the most prevalent histological subtype (54% of cases) among individuals aged 15 years and above (data not shown). Our results are consistent with similar studies carried out in other African settings such as Uganda, Malawi, Kenya, and South Africa ^21, 43–46^, confirming the crucial role of HIV-induced immunosuppression in the development of conjunctiva cancer. HIV is an established cause of conjunctiva cancer, based on the results of a number of case–control and cohort studies ^6^. However, the underlying infectious carcinogen remains unknown, with some recent evidence suggesting a role for EBV ^47, 48^.

For other cancers with limited evidence of causal associations with HIV infection ^6^, we found no significant difference in the odds of developing non-melanoma skin cancer (NMSC), nor liver cancer in PLHIV compared to HIV-negative persons. It is worth noting that there is no established infectious cause of NMSC (as is also the case for conjunctival cancer). As for liver cancer, the majority of current evidence comes from high-income countries where liver cancer is rare and largely caused by hepatitis C virus (HCV), particularly in intravenous drug users ^49^. However, no association has been observed in Africa, where both liver cancer and HIV are prevalent. This could be due to the fact that HBV is the leading cause of liver cancer in Africa ^50^, and the ART used to treat HIV and HBV is the same ^51^. Consequently, PLHIV receive chemoprevention for their HBV-related liver cancer risk, while HBV-positive individuals in the general population may remain undiagnosed and untreated, thereby increasing their likelihood of developing liver cancer.

This study presents some limitations that should be acknowledged, of which the primary one is sub-optimal identification of HIV status in the cancer registry, even after complementary linkage with the HIV registry. This limitation tends to pull associations towards the null and to underestimate the size of relative risks (due to misclassification of some HIV-positive cancers in the unknown HIV-status group who were considered to be HIV-negative). Nevertheless, our comparisons suggested that a large majority of cancers with unknown HIV status were likely to be HIV-negative (with the exception, perhaps, of KS cases of unknown HIV status, see above), and identification of all previously established associations highlights the plausibility of our approach. Furthermore, our findings of HIV associations were not materially affected by restricting analyses to individuals with confirmed HIV status. While we report the proportion of cancers that were known to be diagnosed in PLHIV (helping to order cancer sites by strength of HIV association), these proportions are under-estimates, and cannot be used to directly infer attributable fractions of cancer due to HIV. Finally, while our current data covers the period of 12 years (2007–2018), we were unable to assess trends in HIV-related cancers over time as numerous factors could potentially confound cancer ascertainment in this population. For example, Rwanda has made substantial investments in its healthcare sector, such as constructing healthcare infrastructure, enhancing diagnostic capabilities, training more pathologists, and implementing universal health coverage. These efforts have greatly contributed to the rise in the number of individuals receiving treatment at various healthcare facilities. Thus, the trend in cancer diagnoses over time may signal the impact of those changes rather than the actual changes in cancer incidence. However, with respect to the robustness of our findings on associations with HIV, the completeness of the cancer registry for any given cancer site is not expected to be differential by HIV status. There might be some confounding by risk factors not captured in our registry-based study, such as smoking and alcohol consumption; however, these exposures are not known to be strong risk factors for the cancers associated with HIV in our analysis, and no associations were seen for tobacco- and or alcohol-related cancers (e.g. lung, liver and head and neck cancers – see above). Lastly, whilst information on HIV status was obtained at the time of cancer diagnosis, indicating HIV infection preceded cancer diagnosis, we cannot determine how long participants acquired HIV before cancer onset.

In summary, our study highlights that following the significant expansion of ART coverage in Rwanda, HIV infection is associated with a wide variety of cancers. Our findings are likely applicable to other sub-Saharan African settings with similar ART coverage and can inform further efforts to alleviate the burden of cancer among this population. For instance, targeted cancer prevention initiatives such as screening for HPV-related cancers and expansion of HPV vaccination programs are crucial steps in addressing this issue. Moreover, incorporating HIV testing into the routine medical care of cancer patients can greatly aid in assessing the burden of cancer among PLHIV in the future. This information will be valuable in predicting how HIV control measures will affect the prevalence of cancer in the coming years and determining whether cancer management strategies need to be adjusted based on a patient’s HIV status.

## Supplementary Material

Supinfo

## Figures and Tables

**Table 1.** Characteristics of cancer cases by HIV status in the cancer registry between 2007 and 2018 in Rwanda.

Parameter	All n (%)	HIV negative n (%)	Unknown n (%)	HIV positive^1^ n (%)
**Total**	17679 (100)	5813 (32.9)	10636 (60.1)	1230 (7.0)
**Gender**				
Females	10534 (59.6)	3713 (63.9)	6054 (56.9)	767 (62.4)
Males	7145 (40.4)	2100 (36.1)	4582 (43.1)	463 (37.6)
**Age at diagnosis (years)**				
** Median (IQR)**	50 (35–62)	50 (33–61)	52 (36–64)	43 (34–50)
<15	1463 (8.3)	658 (11.3)	770 (7.2)	35 (2.9)
15–24	1055 (6.0)	345 (5.9)	643 (6.1)	67 (5.4)
25–34	1867 (10.6)	546 (9.4)	1095 (10.3)	226 (18.4)
35–44	2541 (14.4)	803 (13.8)	1387 (13.0)	351 (28.6)
45–54	3509 (19.8)	1135 (19.5)	2034 (19.1)	340 (27.6)
55–64	3477 (19.7)	1224 (21.1)	2093 (19.7)	160 (13.0)
65+	3767 (21.3)	1102 (18.9)	2614 (24.6)	51 (4.2)
**Place of residence**				
Kigali City	4380 (24.8)	1111 (19.1)	2805 (26.4)	464 (37.7)
Southern Province	4302 (24.3)	1268 (21.8)	2810 (26.4)	224 (18.2)
Eastern Province	3831 (21.7)	1297 (22.3)	2305 (21.7)	229 (18.6)
Western Province	2810 (15.9)	1090 (18.7)	1531 (14.4)	189 (15.4)
Northern Province	2356 (13.3)	1047 (18.0)	1185 (11.1)	124 (10.1)
**Year of cancer diagnosis**				
2007	499 (2.8)	38 (0.6)	417 (3.9)	44 (3.6)
2008	519 (2.9)	42 (0.7)	436 (4.1)	41 (3.3)
2009	666 (3.8)	51 (0.9)	556 (5.2)	59 (4.8)
2010	680 (3.8)	80 (1.4)	555 (5.2)	45 (3.7)
2011	895 (5.1)	193 (3.3)	640 (6.0)	62 (5.0)
2012	1599 (9.0)	438 (7.5)	1035 (9.7)	126 (10.2)
2013	1946 (11.0)	605 (10.4)	1186 (11.2)	155 (12.6)
2014	2002 (11.3)	720 (12.4)	1144 (10.8)	138 (11.2)
2015	1938 (10.9)	788 (13.6)	1035 (9.7)	115 (9.4)
2016	2069 (11.7)	841 (14.5)	1090 (10.3)	138 (11.2)
2017	2375 (13.4)	1029 (17.7)	1186( 11.2)	160 (13.0)
2018	2491 (14.1)	988 (17.0)	1356 (12.7)	147 (11.9)
**Method of diagnosis**				
Histopathology	12843 (72.7)	4755 (81.8)	7180 (67.5)	908 (73.8)
Clinical only	4754 (26.9)	1058 (18.2)	3378 (31.8)	318 (25.9)
Missing	82 (0.4)	0 (0.0)	78 (0.7)	4 (0.3)

1 Includes the cancers re-classified as HIV-positive from HIV unknown (n=181) or HIV-negative (n=91) by linkage with HIV registry (see Supplementary table 3).

**Table 2.** Spectrum of cancer in women living with and without HIV in the cancer registry between 2007 and 2018

Cancer type^1^	ICD-10 Code	All (n=10,534)		By HIV status						% known HIV Positive
				Negative (n=3,713)		Unknown (n=6,054)		Positive^2^ (n=767)		
		**n**	**%** ^$^	**n**	**%** ^$^	**n**	**%** ^$^	**N**	**%** ^$^	**%** *
Kaposi sarcoma	C46	133	1.3	11	0.3	40	0.7	82	10.7	61.7
Vulva	C71	101	1.0	23	0.6	55	0.9	23	3.0	22.8
Hodgkin lymphoma	C81	116	1.1	57	1.5	42	0.7	17	2.2	14.7
Eye	C69	187	1.8	67	1.8	94	1.6	26	3.4	13.9
Non-Hodgkin lymphoma	C82–C88, C96	337	3.2	108	2.9	183	3	46	6.0	13.6
Cervix uteri	C53	2285	21.7	883	23.8	1138	18.8	264	34.4	11.6
Anus	C21	30	0.3	9	0.2	19	0.3	2	0.3	6.7
Placenta	C58	195	1.9	90	2.4	93	1.5	12	1.6	6.2
Bladder	C67	33	0.3	9	0.2	22	0.4	2	0.3	6.1
Breast	C50	2107	20.0	825	22.2	1168	19.3	114	14.9	5.4
Bone(osteosarcoma)	C40–C41	277	2.6	84	2.3	180	3.0	13	1.7	4.7
Non-melanoma of skin	C44	211	2.0	47	1.3	154	2.5	10	1.3	4.7
Endometrium	C54–C55	241	2.3	74	2.0	156	2.6	11	1.4	4.6
Lung	C34	175	1.7	63	1.7	104	1.7	8	1.0	4.6
Esophagus	C15	68	0.7	12	0.3	53	0.9	3	0.4	4.4
Colorectum	C18–C20	369	3.5	120	3.2	234	3.9	15	2.0	4.1
Unspecified primary site	–	478	4.5	137	3.7	323	5.3	18	2.4	3.8
Liver	C22	382	3.6	106	2.9	262	4.3	14	1.8	3.7
Stomach	C16	771	7.3	233	6.3	510	8.4	28	3.7	3.6
Vagina	C52	28	0.3	11	0.3	16	0.3	1	0.1	3.6
Brain, central nervous system	C70–C72	200	1.9	51	1.4	142	2.4	7	0.9	3.5
Connective and soft tissue	C49	205	2.0	57	1.5	141	2.3	7	0.9	3.4
Thyroid	C73	119	1.1	29	0.8	86	1.4	4	0.5	3.4
Leukemia	C91–C95	375	3.6	206	5.6	157	2.6	12	1.6	3.2
Head and neck	C00–C14 & C30–32	313	3.0	88	2.4	215	3.6	10	1.3	3.2
Kidney	C64–C66; C68	196	1.9	97	2.6	94	1.6	5	0.7	2.6
Pancreas	C25	77	0.7	24	0.7	51	0.8	2	0.3	2.6
Melanoma of skin	C43	173	1.6	47	1.3	122	2.0	4	0.5	2.3
Ovary	C56	352	3.3	145	3.9	200	3.3	7	0.9	2.0

1 Shown in descending order of HIV prevalence.

2 Includes cancers re-classified as HIV-positive from HIV unknown (n=174) or HIV-negative (n=91) by linkage with HIV registry (see Supplementary table 3).

$ Column percentages.

* Row percentages (assuming HIV-unknown to be HIV-negative).

**Table 3.** Spectrum of cancer in men living with and without HIV in the cancer registry between 2007 and 2018

Cancer type^1^	ICD-10 Code	All (n=7,145)		By HIV status						% known HIV Positive
				Negative (n=2,100)		Unknown (n=4,582)		Positive^2^ (n=463)		
		n	%^$^	n	%^$^	n	%^$^	n	%^$^	%*
Kaposi sarcoma	C46	265	3.7	32	1.5	65	1.4	168	36.3	63.4
Anus	C21	22	0.3	4	0.2	14	0.3	4	0.9	18.2
Penis	C60	260	3.6	65	3.1	162	3.5	33	7.1	12.7
Eye	C69	215	3.0	88	4.2	104	2.3	23	5.0	10.7
Non-Hodgkin lymphoma	C82–C88, C96	478	6.7	163	7.8	272	5.9	43	9.3	9.0
Breast	C50	114	1.6	35	1.7	70	1.5	9	1.9	7.9
Hodgkin lymphoma	C81	174	2.4	64	3.1	97	2.1	13	2.8	7.5
Lung	C34	200	2.8	62	3.0	126	2.8	12	2.6	6.0
Head and neck	C00–C14 & C30–32	504	7.1	165	7.9	317	6.9	22	4.8	4.4
Liver	C22	485	6.8	157	7.5	307	6.7	21	4.5	4.3
Melanoma	C43	96	1.3	20	1.0	72	1.6	4	0.9	4.2
Pancreas	C25	73	1.0	26	1.2	44	1.0	3	0.7	4.1
Leukemia	C91–C95	422	5.9	220	10.5	186	4.1	16	3.5	3.8
Bone(osteosarcoma)	C40–C41	274	3.8	75	3.6	189	4.1	10	2.2	3.6
Colorectum	C18–C20	346	4.8	116	5.5	218	4.8	12	2.6	3.5
Connective and soft tissue	C49	181	2.5	44	2.1	131	2.9	6	1.3	3.3
Stomach	C16	748	10.5	219	10.4	506	11	23	5.0	3.1
Non-melanoma of skin	C44	170	2.4	40	1.9	125	2.7	5	1.1	2.9
Thyroid	C73	34	0.5	11	0.5	22	0.5	1	0.2	2.9
Testis	C62	38	0.5	8	0.4	29	0.6	1	0.2	2.6
Unspecified primary site	–	499	7.0	136	6.5	353	7.7	10	2.2	2.0
Esophagus	C15	157	2.2	50	2.4	104	2.3	3	0.7	1.9
Bladder	C67	53	0.7	14	0.7	38	0.8	1	0.2	1.9
Brain & central nervous system	C70–C72	213	3.0	45	2.1	164	3.6	4	0.9	1.9
Prostate	C61	988	13.8	181	8.6	792	17.3	15	3.2	1.5
Kidney	C64–C66; C68	136	1.9	60	2.9	75	1.6	1	0.2	0.7

1 Shown in descending order of HIV prevalence.

2 Includes cancers re-classified as HIV-positive from HIV unknown (n=174) or HIV-negative (n=91) by linkage with HIV registry (see Supplementary table 3).

$ Column percentages.

* Row percentages (assuming HIV-unknown to be HIV-negative)

## Data Availability

The cancer registry and HIV data utilized in this study were obtained from the Rwanda Biomedical Center and the Ministry of Health of Rwanda. Data sharing is governed by the policies and permissions established by these institutions. To request access to the data or make inquiries concerning data sharing, interested parties are encouraged to directly contact the Rwanda Biomedical Center at info@rbc.gov.rw. Further information is available from the corresponding author upon request.

## Novelty and Impact

Understanding cancer risk among people living with HIV (PLHIV) is highly relevant for cancer control in sub-Saharan Africa. Employing data from the Rwanda population-based cancer registry, this study reveals a strong link between HIV infection and a diverse spectrum of cancers, even in an era of high antiretroviral therapy (ART) coverage. These findings have broader implications for other sub-Saharan African settings and can inform future strategies to address the burden of cancer among PLHIV.

### Novelty & Impact Statement:

Understanding cancer risk among people living with HIV (PLHIV) is highly relevant for cancer control in sub-Saharan Africa, where HIV burden is exceptionally high. The authors of this study utilized data from the Rwanda population-based cancer registry to compare cancer incidence among PLHIV and HIV-negative individuals. PLHIV accounted for 7 percent of cancers diagnosed between 2007 and 2018 in Rwanda. HIV infection was linked to a diverse spectrum of cancers, even in an era of high antiretroviral therapy coverage. The findings offer insight into cancer burden among PLHIV and are relevant to understanding cancer incidence in other sub-Saharan African settings.

## List of abbreviations

ART: Antiretroviral therapy
CI: Confidence intervals
EBV: Epstein-Barr virus
HBV: Hepatitis B virus
HCC: Hepatocellular carcinoma
HCV: Hepatitis C virus
HHV: Human herpesvirus
HIV: Human immunodeficiency virus
HL: Hodgkin lymphoma
HPV: Human papillomavirus
IARC: International Agency for Research on Cancer
ICD-O: International Classification of Diseases for Oncology
IQR: Interquartile range
KS: Kaposi’s sarcoma
MLWH: Men living with HIV
MRS: Medical Record System
NHL: Non-Hodgkin lymphoma
NIH: National Institutes of Health
NMSC: Non-melanoma skin cancer
OR: Odds ratios
PLHIV: People living with human immunodeficiency virus
RNEC: Rwanda National Ethics Committee
SALI: Software for Automated Linkage
SCC: Squamous cell carcinoma
SSA: Sub-Saharan Africa
UICC: Union for International Cancer Control
WE-ACTx: Women’s Equity in Access to Care and Treatment
WHO: World Health Organization
WLWH: Women living with HIV
