# Supplementary material for: Cancer risk among people living with HIV in Rwanda from 2007 to 2018
Source: Int J Cancer. Author manuscript; Available in PMC 2025 Jun 17. (PMC12173455; doi:10.1002/ijc.35091)
Supplement: Supinfo [file NIHMS2007380-supplement-Supinfo.pdf]

Cancer risk among people living with HIV in Rwanda from 2007 to 2018

JC Dusingize, Gad Murenzi, Benjamin Muhoza, Lydia Businge, Eric Remera, Francois Uwinkindi, Marc Hagenimana, Gallican Rwibasira, Sabin Nsanzimana, Philip E. Castle, Kathryn Anastos, Gary Clifford

Table of contents

Supplementary Table 1. Hospitals/Clinics covered by the cancer registry..2

Supplementary Table 2. The distribution of cancer cases linked with HIV registry, according to original HIV status in the cancer registry...3

Supplementary Table 3. Association of HIV infection with specific cancers by age group in Rwanda, 2007–2018...4

Supplementary Table 4. Association of HIV infection with specific cancers diagnosed in Rwanda, 2007-2018, restricted to people with confirmed HIV status only. 5

Supplementary Table 5. Association of HIV infection with specific cancers diagnosed in Rwanda, 2007-2018, after exclusion of all HIV-associated cancers[1]..6

Supplementary Table 6. Association of HIV infection with specific cancers diagnosed in Rwanda, 2013-2018. 7

Supplementary Table 1. Hospitals/Clinics covered by the cancer registry

SN	HOSPITAL/CLINIC NAME	Province
1	University Teaching Hospital of Kigali	City of Kigali
2	King Faisal Hospital	City of Kigali
3	University Teaching Hospital of Butare	South
4	MBC hospital	City of Kigali
5	Butaro District Hospital	East
6	Rwinkwavu District Hospital	East
7	Rwanda Military Hospital	City of Kigali
8	Muhima District Hospital	City of Kigali
9	Masaka District Hospital	City of Kigali
10	Kibagabaga District Hospital	City of Kigali
11	Baho International Hospital	City of Kigali
12	Kabgayi District Hospital	South
13	Hospital La Croix du South	City of Kigali
14	Lancent	City of Kigali
15	Kacyiru District Hospital	City of Kigali
16	Clinic La Medicale	City of Kigali
17	Polyclinic Saint Jean	City of Kigali
18	Legacy Clinic	City of Kigali
19	Clinic Imagerie	City of Kigali

Supplementary Table 2. The distribution of cancer cases linked with HIV registry, according to original HIV status in the cancer registry.

	**Original HIV status in the cancer registry**		
	**Negative N/ n linked (%)**	**Positive N/ n linked (%)**	**Unknown N/ n linked (%)**
**All**	5,813 / 91 (1.6%)	1,230 / 77 (6.3%)	10,636 / 174 (1.7%)
**By cancer type**			
Kaposi sarcoma	43 / 1 (2.3)	250 / 18 (7.2)	105 / 6 (5.7)
Cervix uteri	883 / 19 (2.2)	264 / 13 (4.9)	1,138 / 32 (2.8)
Non-Hodgkin lymphoma	271 / 6 (2.2)	89/ 9 (10.1)	455 / 20 (4.4)
Hodgkin lymphoma	121 / 0 (0.0)	30 / 5 (20.0)	139 / 5 (3.6)
Penis	65 / 4 (6.2)	33 / 0 (0.0)	162 / 5 (0.6)
Anus	13 / 0 (0.0)	6 / 0 (0.0)	33 / 1 (3.0)
Eye	155/ 1 (0.6)	49/ 7 (14.3)	198/ 6 (3.3)
Vulva	23/ 0 (0.0)	23/ 3 (13.0)	55/ 1 (1.8)
All others	4,239/ 60 (1.4)	486/ 22 (4.5)	8,351/ 98 (1.2)
**By place of residence**			
Kigali	1,111/ 40 (3.6)	464/ 48 (10.3)	2,805/ 87 (3.1)
Outside Kigali	4,702/ 51 (1.1)	766/ 29 (3.8)	7,831/ 87 (1.1)

Supplementary Table 3. Association of HIV infection with specific cancers by age group in Rwanda, 2007–2018

Cancer type			By age group (in years)			
	All age groups N (% known HIV+)		<34 N (% known HIV+)	35-44 N (% known HIV+)	45-54 N (% known HIV+)	55+ N (% known HIV+)
**KS**	398 (62.8)		163 (65.1)	101 (71.3)	79 (59.5)	55 (45.6)
**Cervix**	2,285 (11.6)		190 (15.3)	458 (21.4)	729 (12.2)	908 (5.3)
**NHL**	815 (10.9)		419 (9.1)	104 (22.1)	101 (13.9)	191 (7.3)
**HL**	290 (10.3)		199 (6.3)	30 (23.3)	31 (29.0)	30 (6.7)
**Penis**	260 (12.7)		26 (23.1)	33 (27.3)	39 (28.2)	162 (4.3)
**Anus**	52 (11.5)		7 (14.3)	5 (0.0)	17 (29.4)	23 (0.0)
**Eye**	402 (12.2)		226 (6.8)	55 (27.3)	38 (31.6)	43 (9.3)
**Vulva**	101 (22.8)		22 (36.4)	20 (35.0)	27 (29.6)	32 (0.0)
**All others[1]**	13,076 (3.7)		3,093 (3.6)	1,735 (6.9)	2,448 (5.9)	5,800 (1.9)

[1]All others includes all the non-HIV-associated cancers not named above and presented in Supplementary Table 5.

Supplementary Table 4. Association of HIV infection with specific cancers diagnosed in Rwanda, 2007-2018, restricted to people with confirmed HIV status only.

**Cancer type**	**Women (n= 4480)**	**Men (n= 2563)**	**All (n= 7043)**
	**Odds Ratio[1] (95% CI)**	**Odds Ratio (95% CI)**	**Odds Ratio (95% CI)**
Kaposi sarcoma	**35.65 (17.82-71.29)**	**31.02 (20.57-46.78)**	**30.91 (21.75-43.93)**
Vulva	**5.51 (2.9-10.36)**	-	**5.51 (2.9-10.36)**
Penis	-	**3.46 (2.20-5.44)**	**3.46 (2.20-5.44)**
Eye	**2.55 (1.53-4.23)**	**1.77 (1.05-2.99)**	**2.10 (1.46-3.03)**
Cervix uteri	**2.24 (1.87-2.68)**	-	**2.24 (1.87-2.68)**
Anus	1.54 (0.30-7.77)	4.06 (0.93-7.74)	2.48 (0.88-6.94)
Non-Hodgkin lymphoma	**1.88 (1.30-2.74)**	**1.21 (0.83-1.75)**	**1.51 (1.16-1.96)**
Hodgkin lymphoma	1.61 (0.90-2.88)	1.03 (0.54-1.98)	1.30 (0.85-2.01)
Breast	0.66 (0.53-0.82)	1.39 (0.65-2.99)	0.69 (0.56-0.86)
Lung	0.51 (0.23-1.11)	0.97 (0.51-1.87)	0.73 (0.44-1.20)
Endometrium	0.75 (0.39-1.45)	-	0.75 (0.39-1.45)
Bone(osteosarcoma)	0.71 (0.38-1.29)	0.80 (0.40-1.60)	0.76 (0.48-1.20)
Liver (all histological subtypes)	0.55 (0.30-0.99)	0.46 (0.28-0.76)	0.48 (0.33-0.71)
Liver (HCC only)	0.54 (0.26-1.12)	0.53 (0.29-0.96)	0.52 (0.33-0.83)
Non-melanoma of skin	1.49 (0.73-3.08)	0.75 (0.29-1.96)	1.09 (0.62- 1.93)
Head and neck	0.56 (0.28-1.09)	0.71 (0.44-1.13)	0.66 (0.44-0.96)
Colon-rectum	0.70 (0.41-1.18)	0.60 (0.35-1.04)	0.65 (0.44-0.94)
Vagina	0.48 (0.06-3.94)	-	0.48 (0.06-3.94)
Leukemia	0.13 (0.04-0.42)	0.17 (0.05-0.55)	0.15 (0.06-0.34)
Bladder	1.67 (0.32-8.58)	0.37 (0.04-2.92)	0.75 (0.21-2.61)
Connective and soft tissue	0.70 (0.29-1.71)	0.63 (0.28-1.42)	0.67 (0.37-1.22)
Stomach	0.68 (0.45-1.03)	0.49 (0.31-0.78)	0.58 (0.43-0.79)
Pancreas	0.49 (0.14-1.73)	0.34 (0.07-1.56)	0.42 (0.16-1.10)
Thyroid	0.66 (0.22-1.96)	0.36 (0.04-3.03)	0.57 (0.21-1.49)
Melanoma of skin	0.51 (0.18-1.44)	0.95 (0.31-2.93)	0.66 (0.30-1.41)
Esophagus	1.44 (0.36-5.72)	0.30 (0.09-1.00)	0.49 (0.21-1.17)
Brain & central nervous system	0.63 (0.28-1.42)	0.70 (0.29-1.71)	0.67 (0.37-1.22)
Testis	-	0.81 (0.10-6.68)	0.81 (0.10-6.68)
Ovarian	0.23 (0.11-0.50)	-	0.23 (0.11-0.50)
Kidney	0.44 (0.17-1.16)	0.14 (0.01-1.12)	0.32 (0.13-0.77)
Prostate	-	0.63 (0.35-1.12)	0.63 (0.35-1.12)
Oropharynx	0.27 (0.10-0.76)	0.77 (0.45-1.31)	0.57 (0.35-0.91)

[1]Odds ratios determined using logistic regression models adjusting for age, gender (where applicable), place of residence and year of cancer diagnosis.

Supplementary Table 5. Association of HIV infection with specific cancers diagnosed in Rwanda, 2007-2018, after exclusion of all HIV-associated cancers[1]

Cancer type	Women (n= 7,345)	Men (n= 5,731)	All (n= 13,076)
	Odds Ratio[2] (95% CI)	Odds Ratio (95% CI)	Odds Ratio (95% CI)
Leukemia	0.76 (0.42-1.39)	1.38 (0.80-2.38)	1.04 (0.70-1.55)
Breast	1.44 (1.14-1.83)	2.76 (1.37-5.57)	1.60 1.27-2.02)
Ovarian	0.48 (0.23-1.04)	-	0.48 (0.23-1.04)
Vagina	1.00 (0.13-7.50)	-	1.00 (0.13-7.50)
Endometrium	1.07 (0.58-2.00)	-	1.07 (0.58-2.00)
Prostate	-	0.67 (0.38-1.18)	0.67 (0.38-1.18)
Testis	-	0.79 (0.11- 5.86)	0.79 (0.11- 5.86)
Head and neck	0.78 (0.41-1.48)	0.74 (0.41-1.15)	1.20 (0.83-1.73)
Oropharynx	0.41 (0.15-1.10)	0.82 (0.49-1.35)	1.09 (0.69-1.70)
Esophagus	1.33 (0.41-4.32)	0.66 (0.21-2.11)	0.87 (0.38-1.98)
Stomach	1.07 (0.71-1.59)	1.08 (0.69-1.69)	1.05 (0.78-1.42)
Liver (all histological subtypes)	0.96 (0.55-1.67)	1.47 (0.92-2.35)	1.18 (0.83-1.68)
Liver (HCC only)	0.92 (0.47-1.82)	1.52 (0.87-2.66)	1.18 (0.77-1.81)
Colon-rectum	1.12 (0.66-1.91)	1.10 (0.61-2.01)	1.08 (0.73-1.60)
Melanoma of skin	0.71 (0.26-1.93)	1.44 (0.52-3.97)	0.94 (0.46-1.91)
Non-melanoma of skin	1.23 (0.64-2.36)	0.95 (0.38-2.35)	1.11 (0.66-1.88)
Bone(osteosarcoma)	1.19 (0.67-2.12)	1.26 (0.65-2.45)	1.22 (0.79-1.88)
Thyroid	0.76 (0.28-2.10)	0.92 (0.12-6.80)	0.79 (0.32-1.95)
Brain & central nervous system	0.81 (0.38-1.74)	1.04 (0.45-2.40)	0.91 (0.52- 1.59)
Bladder	1.66 (0.39-7.10)	0.62 (0.08-4.49)	1.03 (0.32-3.30)
Lung	0.75 (0.37-1.56)	1.08 (0.56-1.93)	0.85 (0.55-1.40)
Kidney	0.98 (0.39-2.46)	0.30 (0.04-2.22)	0.70 (0.31-1.61)
Pancreas	0.77 (0.19-3.17)	1.45 (0.45-4.68)	1.05 (0.43-2.59)
Connective and soft tissue	0.81 (0.38-1.74)	1.04 (0.45-2.40)	0.91 (0.52-1.60)

[1]Excluded those cancers significantly associated with HIV infection in Table 4, namely NHL, HL, penis, eye, cervix uteri, anus, Kaposi's sarcoma and vulva.
[2]Odds ratios determined using logistic regression models adjusting for age, gender (where applicable), place of residence and year of cancer diagnosis.

Supplementary Table 6. Association of HIV infection with specific cancers diagnosed in Rwanda, 2013-2018.

Cancer type	Women (n=7,781)	Men (n=5,040)	All (n=12,821)
	**Odds Ratio[1] (95% CI)**	**Odds Ratio (95% CI)**	**Odds Ratio (95% CI)**
Kaposi sarcoma	**17.94 (10.36-31.05)**	**25.05 (17.18-36.52)**	**21.45 (15.74-29.22)**
Vulva	**4.180 (2.45-7.12)**	**-**	**4.180 (2.45-7.12)**
Penis	-	**4.19 (2.71-6.49)**	**4.19 (2.71-6.49)**
Eye	**2.72 (1.59-4.63)**	1.91 (0.98-3.69)	**2.33 (1.54-3.52)**
Cervix uteri	**2.55 (2.12-3.06)**	-	**2.55 (2.12-3.06)**
Anus	**1.33 (0.30-5.80)**	3.71 (0.78-17.72)	1.96 (0.68-5.69)
Non-Hodgkin lymphoma	**1.59 (1.05-2.39)**	**1.61 (1.06-2.43)**	**1.61 (1.20-2.15)**
Hodgkin lymphoma	**2.18 (1.13-4.19)**	1.53 (0.75-3.10)	**1.81 (1.12-2.92)**
Breast	0.70 (0.55-0.89)	1.37 (0.62-3.01)	0.73 (0.58-0.92)
Lung	0.58 (0.23-1.44)	1.06 (0.53-2.13)	0.80 (0.46-1.39)
Endometrium	0.57 (0.25-1.31)	-	0.57 (0.25-1.31)
Bone(osteosarcoma)	0.77 (0.41-1.43)	0.78 (0.37-1.61)	0.77 (0.48-1.24)
Liver (all histological subtypes)	0.55 (0.29-1.06)	0.76 (0.43-1.32)	0.65 (0.42-0.98)
Liver (HCC only)	0.55 (0.25-1.19)	0.78 (0.41-1.51)	0.66 (0.40-1.08)
Non-melanoma of skin	0.61 (0.28-1.32)	0.50 (0.18-1.36)	0.56 (0.30-1.03)
Head and neck[2]	0.51 (0.25-1.05)	1.08 (0.67-1.76)	0.82 (0.55-1.22)
Colon-rectum	0.52 (0.26-1.02)	0.75 (0.40-1.39)	0.61 (0.38-0.96)
Vagina	1.19 (0.15-9.37)	-	1.19 (0.15-9.37)
Leukemia	0.53 (0.28-0.98)	0.75 (0.42-1.33)	0.63 (0.41-0.96)
Bladder	1.40 (0.32-6.13)	0.44 (0.06-3.28)	0.77 (0.24-2.49)
Connective and soft tissue	0.43 (0.17-1.05)	0.37 (0.11-1.17)	0.40 (0.20-0.82)
Stomach	0.72 (0.47-1.10)	0.70 (0.44-1.14)	0.70 (0.51-0.96)
Pancreas	0.94 (0.22-4.01)	0.43 (0.05-3.21)	0.65 (0.20-2.10)
Thyroid	0.51 (0.18-1.39)	NA	0.42 (0.15-1.15)
Melanoma of skin	0.25 (0.06-1.04)	0.53 (0.13-2.21)	0.34 (0.12-0.94)
Esophagus	1.18 (0.35-3.94)	0.32 (0.07-1.32)	0.57 (0.23-1.41)
Brain & central nervous system	0.43 (0.19-0.99)	0.19 (0.04-0.79)	0.33 (0.16-0.68)
Testis	-	0.57 (0.07-4.24)	0.57 (0.07-4.24)
Ovarian	0.19 (0.07-0.52)	-	0.19 (0.07-0.52)
Kidney	0.61 (0.22-1.70)	0.22 (0.03-1.63)	0.44 (0.18-1.10)
Prostate	-	0.41 (0.22-0.74)	0.41 (0.22-0.74)
Oropharynx	0.25 (0.07-0.78)	1.28 (0.74-2.20)	0.75 (0.46-1.22)

[1]Odds ratios determined using logistic regression models adjusting for age, gender (where applicable), place of residence and year of cancer diagnosis.
[2]Head and neck includes oropharynx.
NA: The model did not converge due to the small number of cases.
